# Assessment of the Knowledge of Chronic Kidney Disease and Anemia Among University Students in Ghana: A Cross-Sectional Study

**DOI:** 10.1155/tswj/9993948

**Published:** 2025-04-07

**Authors:** Israel A. Sarfo, Benedicta Boakye, Henrietta Eshun, Emmanuel Jingbeja, Abigail Asmah-Brown, Patrick Adu, Joseph Boachie

**Affiliations:** ^1^Department of Medical Laboratory Sciences, University of Cape Coast, Cape Coast, Ghana; ^2^Department of Epidemiology and Disease Control, University of Ghana, Accra, Ghana; ^3^Department of Laboratory, Cape Coast Teaching Hospital, Cape Coast, Ghana

**Keywords:** anemia, chronic kidney disease, diabetes, hemoglobinopathies, hypertension, iron deficiency anemia

## Abstract

**Background:** Chronic kidney disease (CKD) is typically associated with anemia, with both implicated in global mortalities and morbidities. Adequate knowledge about these conditions might help individuals to prevent and/or manage them effectively. This study was aimed at assessing the knowledge of CKD and anemia regarding their causes, risk factors, and preventive practices among undergraduate students.

**Methods:** The study was a cross-sectional design conducted from July 2023 to September 2023, involving 267 students pursuing either health-related or nonhealth-related programs. A structured questionnaire was administered to participants to assess their knowledge of CKD and anemia and was analyzed.

**Results:** An overwhelming majority, 208 (77.9%), demonstrated a good level of general knowledge of CKD, whereas an even higher proportion, 215 (80.5%), had a good level of general knowledge of anemia. Also, the bulk of the participants, 222 (83.1%), showed a good level of knowledge of the relationship between CKD and anemia. There was a significant relationship between a student's faculty and general CKD knowledge (*p* < 0.001). Participants in health-related faculties and in the third/fourth year significantly had good knowledge of CKD than those in nonhealth-related faculties and in the first/second year of studies. With regards to anemia, individuals aged 20 years and beyond had good knowledge of anemia than teenage students. There was also a significant relationship between a student's faculty and general knowledge of anemia (*p* < 0.001), such that participants in health-related faculties were about 99% less likely to have poor knowledge of anemia than those in nonhealth-related faculties [AOR = 0.01 (0.00, 0.007)].

**Conclusion:** Students with good knowledge of CKD, anemia, and/or their interrelationships were the majority. However, students in health-related faculties significantly had good knowledge of both CKD and anemia than their counterparts in nonhealth-related faculties. Health-related courses that would enlighten students in nonhealth-related faculties should be promoted.

## 1. Introduction

Chronic kidney disease (CKD), characterized by a defect in kidney function and structure, is an independent risk factor for anemia and subsequently cardiovascular disease (CVD), posing a global health burden [1]. The paramount role of the kidneys in the production of erythropoietin is hampered in kidney diseases. Hemoglobin levels, therefore, are reported to be lower in patients with CKD [2]. The appearance of anemia in CKD might be the result of insufficiency of erythropoietin due to a decline in renal mass, inhibition of erythropoiesis by uraemia, an abridged survival of red cells, and/or dysregulation in iron homeostasis resulting in iron deficiency [3].

There appear to be limited studies in sub-Saharan Africa on CKD knowledge. In Benin City, Nigeria, less than half (44.7%) of the nonmedical university students were aware of diabetes mellitus, whereas a quarter (25.1%) were aware of hypertension as a key risk factor for CKD [4]. The same study showed that 25.1%, 35.2%, and 39% of the participants had a good level of knowledge, some level of knowledge, and poor knowledge of CKD, respectively [4]. Likewise, among nursing students in different states of the same country, almost eight in 10 and about one in five demonstrated good knowledge of kidney function and CKD, respectively [5].

Correlating the knowledge of CKD with its prevalence, various CKD risk factors including obesity, proteinuria, and glycosuria were reported in 12.2%, 12.4%, and 2.7% of students, respectively, implicating a high risk of CKD development among university students [6]. Whereas some previous evidence [7, 8] showed that nonmodifiable factors such as gender, age, level of education, and personal income of participants were all associated with CKD, others highlighted some modifiable factors, including diabetes, excessive alcohol, obesity, hypertension, and diabetes mellitus, to be associated with CKD [9]. One paramount modifiable factor, knowledge, was proposed to be highly significant in the prevention of disease [10, 11]. Individuals demonstrating much knowledge and awareness of CKD tend to have a healthy lifestyle, hence, reducing CKD risk [12]. It is generally reported that the population's awareness of CKD remains low [13]. This might be the result of insufficient and/or lack of information targeting its risk factors as well as preventive measures, therefore, hampering the effectiveness of intervention strategies [13].

Anemia remains a common complication in CKD, which consequently accounts for other comorbidities. The absence of adequate awareness of CKD and its associated causes, risk factors, and preventive practices has therefore resulted in an increase in its prevalence. Although anemia occurs in CKD, anemia could develop independently of CKD. Adequate knowledge about both conditions, especially knowledge about their preventive practices, helps individuals to adopt healthy lifestyles. Thus, the current study was aimed at exploring the knowledge of CKD and anemia, their causes, risk factors, and preventive practices among undergraduate university students.

## 2. Methods

### 2.1. Study Setting

The study was conducted at the University of Cape Coast (UCC) in Ghana. UCC is located in Cape Coast, the capital city of the Central Region of Ghana. It was established in the year 1962 with a student population comprising regular undergraduates, postgraduates, distance education students, and sandwich students. UCC currently has a total of about 78,485 students, with about 60,406 students categorized as undergraduates and about 5648 being graduate students. The university largely offers a wide range of programs run by either health-related or nonhealth-related faculties.

### 2.2. Study Design

This was a cross-sectional study conducted among regular undergraduate students in the university from July 2023 to September 2023 to assess the knowledge of CKD and anemia, their causes, risk factors, and preventive practices. Awareness and knowledge assessments were done using structured questionnaires.

### 2.3. Study Population

The study involved only regular undergraduate students from either health-related or nonhealth-related faculties. Some health-related faculties include medical laboratory science, nursing, optometry, and nutrition/dietetics, whereas nonhealth-related faculties comprise mathematics, social science, agricultural science, and chemistry.

### 2.4. Inclusion and Exclusion Criteria

#### 2.4.1. Inclusion Criteria

Only consented regular undergraduate students offering either a health-related or nonhealth-related study program were recruited for the study.

#### 2.4.2. Exclusion Criteria

Postgraduate, sandwich, and distance education students were not involved in the study.

### 2.5. Sample Size

The sample size was calculated using Taro Yamane's formula [14] for sample size determination in a finite population as shown in the following:

 $$\begin{array}{c} n=\frac{N}{1+N{\left(e\right)}^{2}} \end{array}$$

where *n* is the sample size, *N* (population size) = 8364, and *e* (margin of error) = 0.05.

$$\begin{array}{cc} \; & n=\frac{8364}{1+8364{\left(0.05\right)}^{2}}\\ n=382 \end{array}$$

The study therefore targeted about 382 students. The number of questionnaires printed for the study was 400 and distributed to 400 students. Out of these, only 267 responded positively by returning fully completed questionnaires. Other students either did not return the questionnaires or submitted partially filled questionnaires. Therefore, the number of students recruited in our study was 267.

### 2.6. Sampling Technique

A convenience sampling technique was used to select programs from both health-related and nonhealth-related departments in the university. A stratified random sampling technique was then adopted to recruit students offering different programs within six departments to obtain equal proportions from each stratum among undergraduate university students studying at UCC.

### 2.7. Data Collection Instrument

The instrument used for the data collection in the study was a structured questionnaire with close-ended questions. Two validated questionnaires [4, 15], previously adopted in similar studies assessing the knowledge of CKD risk factors and preventive practices, were slightly modified and used to develop our questionnaire. The current study ensured that the questionnaire was made as brief as possible to encourage and maximize the responsiveness of participants. The questionnaire items were developed through focus group discussions among the investigators. The questionnaire was tested for face validity and checked for content saturation. Moreover, the validity and reliability of the questionnaire were evaluated by three lecturers at the university who have in-depth knowledge of CKD and anemia.

The questions captured in the questionnaire targeted sociodemographics, general knowledge, causes, risk factors, and prevention of CKD and anemia, as well as knowledge of the relationship that exists between CKD and anemia.

Every correct answer was awarded one point, whereas an incorrect response was awarded zero points. The probable maximum score was 34 points (100%) for CKD knowledge, 35 points (100%) for anemia knowledge, and 10 points (100%) for knowledge of the CKD–anemia interrelation. Also, the probable minimum score was 0 (0%), and this was applicable to CKD, anemia, or both. Finally, the scores were summed up to obtain the overall knowledge score and presented as percentages. The mean and standard deviation of knowledge scores for CKD, anemia, and the CKD–anemia interrelation were computed, respectively. Scores below the mean score for these three subjects were considered poor knowledge, and scores equal to and above the mean scores were considered good knowledge.

The mean of the knowledge score for CKD, anemia, and the CKD–anemia interrelation were 23.19 (SD 6.98), 23.42 (SD 6.23), and 5.27 (SD 2.57), respectively. Categorization of the overall CKD knowledge of participants was therefore similar, with slight modifications, to Bloom's criteria cut-off points as adopted by previous studies, which considered knowledge scores between 60% and 100% as moderate-to-good, whereas knowledge scores < 60% were categorized as poor [16, 17]. In this study, therefore, any score < 23 (< 65.71%) was defined as low knowledge of CKD, whereas high CKD knowledge was ranked at 23–35 (65.7%–100%). Similarly, < 23 (< 63.9%) scores defined a low knowledge of anemia, whereas 23–36 (63.9%–100%) scores indicated high anemia knowledge [18]. For questions on CKD–anemia interrelation, low knowledge was attributed to any score < 5 (< 50%), while > 5 (> 50%) scores constituted high knowledge [15]. Students with low and high levels of knowledge were reported to have poor and good knowledge levels, respectively.

### 2.8. Data Collection Procedure

The participants' study programs, either health-related or nonhealth-related, and the year level of students were conveniently sampled based on their accessibility and availability. The questionnaires were sent to the selected classes for data collection. The investigator explained the purpose of the study to the students, and the written informed consents were taken once the respondents had agreed. The questionnaires were handed to the class monitors to be distributed to the consenting students. Ample time was given to the respondents to answer the questionnaire, as respondents were allowed to send the questionnaire home and return it another day when coming to class.

### 2.9. Ethical Consideration

Ethical approval was sought from the Institutional Review Board (IRB) of the UCC with the Approval ID: UCCIRB/CHAS/2023/02. Also, written informed consent was obtained from participants before being recruited into the study. Participants were at liberty to withdraw from the study at any given time.

### 2.10. Data Analysis

The data was coded, entered into SPSS version 28.0, and analyzed using descriptive statistics. Knowledge of CKD and anemia, their causes, risk factors, and preventive practices were reported as frequencies and percentages. The chi-square test, crude odds ratio (COR), and adjusted odds ratio (AOR) were employed to analyze the association between sociodemographic characteristics and knowledge of students. The COR and AOR, as well as the corresponding 95% confidence interval (CI), were determined using binary logistic regression and multivariate logistic regression, respectively. All probability values less than 0.05 were considered statistically significant.

## 3. Results

### 3.1. Sociodemographic Characteristics

An overwhelming majority of participants, constituting more than half, 150 (56.2%), were aged between 20 and 23 years, whereas the least, 20 (7.5%), of the respondents were aged ≥ 28 years. Again, the majority of respondents were males, 174 (65.2%), in the fourth academic year, 132 (49.4%), and were affiliated with a health-related faculty, 145 (54.3%). Nearly all the participants, 266 (99.6%), had no family history of CKD. Likewise, the majority of the respondents, 257 (96.3%), also had no family history of anemia (Table 1).

### 3.2. Knowledge of Students on CKD, Causes, Risk Factors, and Preventive Practices

The data (Table 2) indicates that an overwhelming majority, 225 (84.3%), of the respondents accept that the ability of the kidney to remove waste substances from the body is reduced in CKD, and likewise, the majority of them, 223 (83.5%), admit that CKD is damage to the kidney. Also, more than half, 185 (69.3%), admit that the size of the kidney is not normal in CKD, whereas about three in four participants, 193 (72.3%), believe that CKD does not occur in the elderly only. The overwhelming majority of participants (59.9%–75.7%) similarly gave the right responses demonstrating knowledge of CKD, regarding its impact on bone integrity to its manifestation with muscle cramps. However, knowledge of the CKD association with urination difficulties was only known by a few, 64 (24.0%), of the respondents.

With regard to the responses of students on the causes of CKD, the majority of the respondents, representing more than half, acknowledged that untreated hypertension, 200 (74.9%); untreated diabetes, 212 (79.4%); enlarged prostate, 180 (67.4%); and kidney stones, 238 (89.1%), are potential causes of CKD.

The data also shows that most of the respondents believe that untreated hypertension, 210 (78.7%); smoking, 233 (87.3%); family history of kidney disease, 233 (87.3%); obesity, 181 (67.8%); older age, 191 (71.5%); untreated diabetes, 209 (78.3%); drinking too little water, 177 (66.3%); excessive alcohol consumption, 230 (86.1%), and heart-related diseases, 192 (71.9%), are risk factors of CKD. Meanwhile, 185 (69.3%) of the respondents are certain that too much water drinking is not a risk factor for CKD.

With regard to CKD prevention, an overwhelming majority of the participants, constituting at least 215 (80.5%) and at most 245 (91.8%), gave the right responses, such as minimal sugar intake and abandoning smoking, respectively.

### 3.3. Knowledge of Students on Anemia, Causes, Risk Factors, and Preventive Practices and the Relationship Between CKD and Anemia

Respondents' knowledge of anemia, the cause, the risk factors, and preventive practices, as well as the knowledge of respondents on how CKD relates to anemia, are shown in Table 3.

The majority of the respondents, 240 (89.9%), admit that anemia is a disease associated with blood, 237 (88.8%) agree that in anemia the body does not make enough red blood cells, 231 (86.5%) agree that anemia reduces blood hemoglobin concentration, and 231 (86.5%) believe that anemia can affect the transport of oxygen to body tissues. Further assessment showed that a minimum of about seven in 10 of the participants, 191 (71.5%), to a maximum of 245 (91.8%), had knowledge of anemia.

Likewise, the majority of the respondents admit that G6PD enzyme deficiency, 206 (77.2%); iron deficiency, 234 (87.6%); vitamin B12 deficiency, 226 (84.6%); folate deficiency, 204 (76.4%); sickle cell disease, 220 (82.4%); CKD, 218 (81.6%); bone marrow disease, 223 (83.5%); liver cirrhosis, 188 (70.4%); malaria, 177 (66.3%); and diabetes, 165 (61.8%), are possible causes of anemia.

More so, the majority of the respondents admit that pregnancy, 207 (77.5%); too much consumption of alcohol, 208 (77.9%); older age, 192 (71.9%); exposure to toxic chemicals, 229 (85.8%); menstruation, 181 (67.8%); intake of some certain drugs, 218 (81.6%); low intake of iron-containing diets, 236 (82.4%); low intake of vitamin B12-containing diets, 225 (84.3%); and low intake of folate-containing diets, 221 (82.8%), are risk factors for anemia.

Further, 214 (80.1%) admit that regular intake of water is a practice that can be adopted in order to avoid anemia, whereas 225 (84.3%) also admit that regular intake of vitamin B12-rich foods is a practice that can be adopted in order to avoid anemia. Some of the respondents admit that frequent blood donation, 160 (59.9%); eating less iron-rich foods, 166 (62.2%); less intake of folate-rich diets, 137 (51.3%); and less intake of vitamin C-rich diets, 160 (59.9%), are not good preventive practices for anemia.

The data also indicates the knowledge of the students about how CKD relates to anemia. About 153 (57.3%) of the respondents admit that CKD has something to do with anemia, 197 (73.8%) believe that iron deficiency anemia (IDA) is a common complication in CKD, 172 (64.4%) admit that CKD patients with anemia usually have a cognitive impairment, 185 (69.3%) admit that CKD patients with anemia can also have high lipid levels in the blood, 180 (67.4%) admit that CKD patients with anemia can also develop stroke, 191 (71.5%) admit that CKD patients with anemia can develop heart muscle disease and can also develop lung diseases, 181 (67.8%) believe that patients on dialysis can develop anemia, 204 (76.4%) believe that regular intake of iron-rich foods can help manage IDA in CKD patients, and finally, 213 (79.8%) of them also believe that regular intake of vitamin-rich foods and folate-rich foods can help reduce the severity of anemia in CKD patients (Table 3).

### 3.4. Summary (Scores) of the General Knowledge Assessment of Participants

The general knowledge scores for CKD ranged from a minimum score of zero to a maximum score of 34. The general knowledge score for anemia ranged from a minimum score of zero to a maximum score of 35. The knowledge score for the relationship between CKD and anemia ranged from a minimum score of zero to a maximum score of 10. The means of knowledge scores for CKD, anemia, and CKD–anemia interrelation were 23.19 (SD 6.98), 23.42 (SD 6.23), and 5.27 (SD 2.57), respectively. Scores below the mean score for these three subjects were considered poor knowledge, and scores equal to and above the mean scores were considered good knowledge.

Only 22.1% of the respondents indicated a poor level of general knowledge of CKD, whereas a greater number, 77.9%, demonstrated a good level of general knowledge of CKD. From the data, 19.5% of the respondents demonstrated a poor level of general knowledge of anemia, but the majority of them, 80.5%, indicated a good level of general knowledge of anemia. Likewise, a few, 16.9%, demonstrated a poor level of knowledge of the relationship between CKD and anemia, but a greater number, 83.1%, showed a good level of knowledge of the relationship between CKD and anemia (Table 4).

### 3.5. Association of Demographic Factors With the Knowledge Level of Participants on CKD

In the bivariate logistic regression analysis, there was no significant relationship between the age groups and general knowledge of CKD (*p* = 0.35), gender, and general CKD knowledge (*p* = 0.14), as well as the student levels and general CKD knowledge (*p* = 0.07). However, there was a significant relationship between faculty and general CKD knowledge (p <0.001). The majority (nearly half) of the participants in health-related faculties had good general knowledge of CKD, whereas the number of participants in nonhealth-related faculties who had poor CKD knowledge was more than twice that of their counterparts in the health-related faculties with poor CKD knowledge (15.4% vs. 6.7%). The multivariate logistic regression analysis showed that the participants in health-related faculties were about 98% less likely to have poor CKD knowledge compared with those in nonhealth-related faculties [AOR = 0.02 (0.00, 0.09)], showing a good significant association (*p* < 0.001) (Table 5). Interestingly, the multivariate logistic regression analysis also showed that the level of study of a participant was independent and significantly associated with CKD knowledge (*p* < 0.001). A higher number of participants in the third year, 72 (27.0%), and fourth year, 98 (36.7%), generally had good CKD knowledge compared with participants in the first and second years. The probability of third- and fourth-year students demonstrating poor CKD knowledge decreases by 83% [AOR = 0.17 (0.03, 1.16)] and 87% [AOR = 0.13 (0.002, 0.59)], respectively (Table 5).

### 3.6. Association of Sociodemographic Factors With the Knowledge of Participants on Anemia

According to the data (Table 6), the bivariate logistic regression analysis showed a significant relationship between the age groups and general knowledge of anemia (*p* = 0.001) and between faculty and general knowledge of anemia (*p* < 0.001). However, there was no significant relationship between gender and general knowledge of anemia (*p* = 0.62) nor the participants' level of study and general knowledge of anemia (*p* = 0.07). The multivariate logistic regression analysis confirmed a significant association between age group distribution and the knowledge of anemia (*p* = 0.012). Generally, the majority of the participants who were 20 years and beyond, constituting about eight in 10 of the participants, had good knowledge of anemia compared with their fewer counterparts within the same age group who demonstrated poor knowledge of anemia. The probability that students of the ages 20–23, 24–27, and ≥ 28 years do have good knowledge of anemia increases by about 15 times [AOR = 15.21 (1.99, 116.38)], 30 times [AOR = 30.82 (3.35, 283.59)], and 47 times [AOR = 47.26 (4.21, 530.21)], respectively. Contrarily, among the teenage group between 17 and 19 years, more of the participants, 11 (4.1%), had poor knowledge of anemia relative to fewer, 10 (3.7%), of them who had good anemia knowledge. Also, the multivariate logistic regression analysis showed a significant association between the faculty of participants and the demonstration of knowledge about anemia (*p* < 0.001). The majority of participants in health-related faculties, 129 (48.3%), had good knowledge of anemia than those in nonhealth-related faculties, 86 (32.2%). Similarly, participants in nonhealth-related faculties who demonstrated poor knowledge of anemia were more than twice the number of their counterparts in health-related faculties with poor anesmia knowledge (36 [13.5%] vs. 16 [6.0%]). Participants in health-related faculties were about 99% less likely to have poor knowledge of anemia compared with those in nonhealth-related faculties [AOR = 0.01 (0.00, 0.007)], showing a good significant association (*p* < 0.001) (Table 6).

## 4. Discussion

The current study was aimed at assessing and evaluating the knowledge of CKD and anemia among undergraduate students in the UCC, Ghana. The incidence of CKD and anemia has greatly increased, and it has been reported that lack of adequate awareness or knowledge of these conditions is one of the major contributing factors since individuals with poor knowledge are unable to adopt healthy lifestyles and measures that will protect them from these conditions. Studies have shown that young people like university students are also at high risk of developing CKD [6], so this study was purposefully conducted to find out whether undergraduate students in UCC are at a higher risk of acquiring CKD and anemia by assessing their knowledge levels about these conditions.

The findings of the current study indicated that the majority of the respondents had good knowledge about CKD (77.9%), anemia (80.5%), and the relationship that exists between the two conditions (83.1%), with few of them showing a poor knowledge level.

### 4.1. Knowledge of CKD, Causes, Risk Factors, and Preventive Practices

The results of the current study indicated that more than half, 208 (77.9%), had good CKD knowledge, but 59 (22.1%) had a poor knowledge level. Similarly, a cross-sectional study that used a smaller sample size and was conducted in South Africa indicated more than half (60.4%) of the 144 university students surveyed had good knowledge of kidney disease [19]. The current study showed that a little over two in 10 (22.1%) of the respondents had poor general knowledge about CKD. Similarly, other studies have reported a low level of CKD knowledge among a small proportion of their participants. A study that was conducted in Malaysia reported that almost one-third of UKM students have below-average knowledge of CKD [18]. Another local study, which used a smaller sample size (108), showed that 43.5% of respondents had below-average knowledge [9], although the study considered only undergraduates in health-related faculties. Different tools and cut-off points were used to measure the CKD knowledge, and this might have accounted for the difference here.

Conversely, there were many studies indicating the populations had poor knowledge of CKD [4, 20]. A study in Nigeria showed that over a third (39.7%) had a low level of knowledge of kidney disease, another third (35.2%) had some knowledge, and a quarter (25.1%) had good knowledge. The sample involved 295 nonmedical third- and fourth-year university students at a government university in Nigeria [4]. The study [4] considered only students in health-related faculties, but our current study considered students from both health-related and nonhealth-related faculties. In 2014, an Iranian community had limited knowledge of the main CKD risk factors, with only 12.7% being aware of unmanaged diabetes and 14.4% aware of untreated hypertension [21]. This finding is in contrast to the findings of the current study. The current study reported a higher proportion of correct answers for untreated diabetes (78.3%) and untreated hypertension (78.7%) as risk factors for CKD. The study [20] also considered people in the Iranian community but not students, whereas the current study only considered undergraduate students, and since students have easy access to knowledge, this might account for the difference.

### 4.2. Knowledge of Anemia, Causes, Risk Factors, and Preventive Practices

The results of the current study indicated that about eight in 10, 215 (80.5%), had good anemia knowledge, whereas almost two in 10, 52 (19.5%), participants had a poor knowledge level. This implied that the majority (more than half) of the respondents demonstrated a good general knowledge of anemia. Most of the studies similar to ours mostly considered IDA, since a deficiency of iron is the most common cause of anemia. A study that was conducted among only female adolescent students reported that more than half (52.4%) of participants exhibited adequate knowledge and 42.7% had a positive attitude toward IDA [22]. However, our study constituted university students who were both males and females. Also, another cross-sectional study conducted among 100 students at Saveetha University in Chennai reported that their fundamental understanding of anemia and hemoglobin is adequate, but participants lacked the expertise in clinical criteria such as anemia diagnosis and therapy [23]. A nonexperimental descriptive comparative research design on 60 adolescent girls also reported that less than half of the participants had poor knowledge, and one-third had moderate knowledge [24]. Conversely, a study has also reported that among the respondents, the knowledge about IDA and nutritional status was about 37% very poor, 23% good, and 40% moderate [25]. The low level of good knowledge among participants in our study might be a result of the difference in the target populations since the earlier study was conducted among adolescents who may not be much privy to health information like university students. A study also found that participants' knowledge was reported as weak, attitude was reported as negative, and practice assessment was reported as bad [26]. The majority of those investigated in the Tabuk Region had little or no understanding of how to prevent IDA or even demonstrated an unreceptive attitude toward the topic [27].

### 4.3. Association Between Sociodemographic Factors and Knowledge Toward CKD, Causes, Risk Factors, and Preventive Practices

The current study indicated that there was no significant association between gender and CKD knowledge. This finding agrees with a study that reported that male and female respondents had no difference regarding their knowledge of CKD [28]. Conversely, numerous studies have reported that there is a significant relationship between gender and level of knowledge of CKD. A study showed that gender had a significant relationship with the level of knowledge [4]. There is a significant association between gender and knowledge level, whereby females had poorer knowledge compared to male patients [29], and this finding might be a result of the population that was considered. Poor knowledge among female patients in the study may reflect their background, as most of them were typically housewives (24.3%) who had a moderate education level (39.8%) and were unemployed (34%) [29]. The current study considered male and female undergraduate students who equally have access to information. Another study showed there is a significant association between gender and the level of knowledge about CKD and that male students were more likely to have below-average CKD knowledge. It also reported below-average CKD knowledge among male students, possibly due to sex dissimilarities in health information-seeking behavior [9].

The current study also indicated that there is no significant relationship between age and the level of knowledge about CKD. Our finding is in contrast to a study that reported that lower knowledge level was related to aging [28]. The current study indicated that there is a significant association between a student's level of study and knowledge about CKD, whereby students in their third/fourth academic year had good CKD knowledge than those in their first/second year. Conversely, a study (unpublished data) conducted in South Africa reported that as participants moved from a low level to a high level in the year of study, there was a decrease in their level of knowledge [19]. We suggest that health literacy programs should be incorporated into the orientation programs organized for newly admitted students to encourage the first-year students to become health conscious from the onset.

Again, the current study indicated a significant association between a participant's faculty and level of knowledge about CKD. The results indicated that participants from nonhealth-related faculties have higher chances of having poor general knowledge about CKD as compared to those from health-related faculties. Similarly, a study has reported that undergraduate students and students from nonhealth-related faculties/institutes were more likely to have below-average CKD knowledge compared to postgraduate students and those at health-related faculties [9]. The poor knowledge about CKD recorded in the current study among participants in nonhealth-related faculties might be due to the exclusion of health-related courses from the curriculum of these faculties. The poor knowledge about CKD recorded in the current study among participants in nonhealth-related faculties might also be due to low health literacy. It is expected that students from nonhealth-related faculties were more likely to have below-average CKD knowledge compared to students from health-related faculties, as learning the pathophysiology of the disease is not part of their curriculum [9]. Students from health-related faculties are future health caregivers, and having good CKD knowledge is crucial and important for managing this complicated health issue [9]. However, it is important to promote and advance CKD knowledge to all students for better disease prevention [9].

### 4.4. Association Between Sociodemographic Factors and Knowledge About Anemia, Causes, Risk Factors, and Preventive Practices

The present study indicated that there is a significant relationship between age groups and knowledge about anemia. The present study recorded that participants aged 20 years and above had higher chances of acquiring adequate and good knowledge about anemia, its causes, risk factors, and preventive practices. Conversely, a Malaysian Health Literacy Survey showed that Malaysians aged 18–24 years had a higher proportion of limited health literacy compared to Malaysians aged 25–39 years [30]. A German study also indicated that respondents in the youngest age group (15–29 years) had lower health literacy scores compared to young adult respondents (30–45 years) [31]. This variation could have been due to the different roles as well as personal and social obligations of the different age groups. However, these previous studies were conducted among the general population, and these postulations need further exploration, as we did not detect any possible interaction of these factors with anemia knowledge. Since it is most likely that teenage students might be either newly admitted and/or in their first/second year of studies, we suggest the intensification of health awareness programs right from the enrolment of students into the university.

The present study indicated that there is a significant association between faculty and knowledge about anemia. The results indicated that participants from nonhealth-related faculties have higher chances of having poor general knowledge about anemia as compared to those from health-related faculties. The poor knowledge about anemia recorded in the current study among participants in nonhealth-related faculties might be due to the exclusion of health-related courses from the curriculum of these faculties. However, it is important to promote and advance anemia knowledge to all students for better disease prevention [9].

## 5. Limitations

Our study has some limitations. This was a single-center, cross-sectional study that used nonprobability sampling. Whereas about 400 questionnaires were issued, over 390 questionnaires were returned; however, only students who completely filled the questionnaires (267) were involved in the study, thus affecting the overall sample size. Causal inferences may not be drawn, and the findings cannot be inferred from the whole student population. Selection bias may be present, where the participating students could be more health conscious and more inclined to answer the questions. There is the possibility of information bias, as the students could have sought the correct answers prior to answering the survey, resulting in an overestimation of CKD knowledge in this study. Also, prior to the study, students were not screened to check the prevalence of CKD and anemia with knowledge, as this might have also contributed to the overestimation of CKD knowledge in this study. Our study also considered only the quantitative aspect without taking into consideration a qualitative study. Participants were therefore not allowed to orally express their thoughts about the questions and explain in detail the reason why they provided such answers.

## 6. Conclusion

Good knowledge regarding CKD and anemia, with their associated causes, risk factors, and preventive practices, was indicated in 77.9% and 80.5% of the respondents, respectively. Good knowledge regarding the relationship between CKD and anemia was indicated in 83.1% of the respondents in our study. Among all the sociodemographic characteristics, only a participant's faculty was found to have a significant association with both general knowledge about CKD and anemia. Health-related courses that will enlighten students in nonhealth-related faculties should be included in their curriculum. Also, health literacy should be given specifically to students in nonhealth-related faculties and generally to all students to educate them and create awareness of these conditions. This will equally benefit both the young students and the aged ones.

## 7. Recommendation

Future studies should consider a qualitative study. Factors that we postulated earlier that would influence knowledge of CKD and anemia, such as health information-seeking behavior and health literacy, should be examined further. Stratified analysis based on program level and faculty type should also be investigated, as the students have different roles and responsibilities that might influence their knowledge level. In addition to university students, all young populations should be provided with adequate knowledge about CKD and anemia. The young population is at a transitional stage during which they develop either healthy or unhealthy habits that will influence their well-being throughout adulthood [32]. Based on our findings, particularly empowering youths with knowledge of the symptoms of CKD development and progression should be emphasized.

## Figures and Tables

**Table 1:** Sociodemographic characteristics of students.

**Characteristics**	**N** ** (%)**
*Age (years)*	
17–19	21 (7.9)
20–23	150 (56.2)
24–27	76 (28.5)
≥ 28	20 (7.5)
*Sex*	
Male	174 (65.2)
Female	93 (34.8)
*Level*	
First year	30 (11.2)
Second year	23 (8.6)
Third year	82 (30.7)
Fourth year	132 (49.4)
*Faculty*	
Health-related	145 (54.3)
Nonhealth-related	122 (45.7)
*Family history of CKD*	
Yes	1 (0.4)
No	266 (99.6)
*Family history of anemia*	
Yes	10 (3.7)
No	257 (96.3)

*Note:* The data is presented as frequencies and percentages, *n* (%).

Abbreviation: CKD, chronic kidney disease.

**Table 2:** Knowledge of students on CKD, causes, risk factors, and preventive practices.

	**Yes, ** **n** ** (%)**	**No, ** **n** ** (%)**
*Knowledge of CKD*		
In CKD, the kidney's capability to eliminate waste substances from the blood is minimized	225 (84.3)^a^	42 (15.7)
CKD indicates injury to the kidney	223 (83.5)^a^	44 (16.5)
In CKD, the kidneys' sizes are unaffected	82 (30.7)	185 (69.3)^a^
CKD occurs only in the elderly	74 (27.7)	193 (72.3)^a^
CKD could hamper the bones' normal integrity within the body	160 (59.9)^a^	107 (40.1)
CKD generally appears with pain in the flank	202 (75.7)^a^	65 (24.3)
CKD generally appears with urination difficulties	203 (76.0)	64 (24.0)^a^
CKD typically manifests with an alteration in urine appearance, color, and odor	196 (73.4)^a^	71 (26.6)
CKD typically demonstrates with the swelling of the ankle and feet	196 (73.4)^a^	71 (26.6)
CKD generally manifests with muscle cramps	186 (69.7)^a^	81 (30.3)
*Knowledge of causes of CKD*		
Diabetes can cause CKD	212 (79.4)^a^	55 (20.6)
Hypertension can cause CKD	200 (74.9)^a^	67 (25.1)
Enlarged prostate can cause CKD	180 (67.4)^a^	87 (32.6)
Kidney stones can cause CKD	238 (89.1)^a^	29 (10.9)
*Knowledge of risk factors of CKD*		
Untreated hypertension	210 (78.7)^a^	57 (21.3)
Excessive smoking	233 (87.3)^a^	34 (12.7)
Family history of kidney disease	233 (87.3)^a^	34 (12.7)
Obesity	181 (67.8)^a^	86 (32.2)
Older age	191 (71.5)^a^	76 (28.5)
Untreated diabetes	209 (78.3)^a^	58 (21.7)
Excessive water drinking	82 (30.7)	185 (69.3)^a^
Habit of little water drinking	177 (66.3)^a^	90 (33.7)
Excessive alcohol consumption	230 (86.1)^a^	37 (13.9)
Heart-related disease	192 (71.9)^a^	75 (28.1)
*Knowledge of prevention of CKD*		
Engage in regular physical exercise	242 (90.6)^a^	25 (9.4)
Avoid the abuse of pain medicines	226 (84.6)^a^	41 (15.4)
Abandon smoking	245 (91.8)^a^	22 (8.2)
Ingest minimal sugar	215 (80.5)^a^	52 (19.5)
Ingest minimal salt	221 (82.8)^a^	46 (17.2)
Do not ingest high-fat foods	220 (82.4)^a^	47 (17.6)
Do not take excessive alcohol	241 (90.3)^a^	26 (9.7)
Regular fruits intake	236 (88.4)^a^	31 (11.6)
Eat vegetables regularly	238 (89.1)^a^	29 (10.9)
Engage in regular kidney check-ups	242 (90.6)^a^	25 (9.4)

*Note:* The data is presented as frequencies and percentages, *n* (%).

Abbreviation: CKD, chronic kidney disease.

^a^Correct answer.

**Table 3:** Knowledge of students on anemia, causes, risk factors, and preventive practices and the relationship between CKD and anemia.

	**Yes, ** **N** ** (%)**	**No, ** **N** ** (%)**
*Knowledge of anemia*		
Anemia is a disease condition affecting the blood	240 (89.9%)^a^	27 (10.1%)
In anemia, the body does not make enough red blood cells	237 (88.8%)^a^	30 (11.2%)
Anemia decreases blood hemoglobin levels	231 (86.5%)^a^	36 (13.5%)
Anemia distracts oxygen transport to body tissues	231 (86.5%)^a^	36 (13.5%)
Anemia could end up in brain dysfunction	199 (74.5%)^a^	68 (25.5%)
Anemia could end up in cardiovascular diseases	210 (78.7%)^a^	57 (21.3%)
Anemia arises when erythropoietin production reduces	200 (74.9%)^a^	67 (25.1%)
Anemia could hamper the function of the lungs	191 (71.5%)^a^	76 (28.5%)
Anemia could manifest in both the young and the old	245 (91.8%)^a^	22 (8.2%)
Anemia could be deadly	217 (81.3%)^a^	50 (18.7%)
*Knowledge of causes of anemia*		
A defect in the G6PD enzyme	206 (77.2%)^a^	61 (22.8%)
A deficiency of iron	234 (87.6%)^a^	33 (12.4%)
Vitamin B12 deficiency	226 (84.6%)^a^	41 (15.4%)
Folic acid (folate) deficiency	204 (76.4%)^a^	63 (23.6%)
Sickle cell disease results in anemia	220 (82.4%)^a^	47 (17.6%)
Chronic kidney disease can lead to anemia	218 (81.6%)^a^	49 (18.4%)
Bone marrow disease can lead to anemia	223 (83.5%)^a^	44 (16.5%)
Cirrhosis of the liver can lead to anemia	188 (70.4%)^a^	79 (29.6%)
Malaria can lead to anemia	177 (66.3%)^a^	90 (33.7%)
Diabetes can result in anemia	165 (61.8%)^a^	102 (38.2%)
*Knowledge of risk factors of anemia*		
Pregnant women are at risk of anemia	207 (77.5%)^a^	60 (22.5%)
Too much consumption of alcohol	208 (77.9%)^a^	59 (22.1%)
Older people are at risk of anemia	192 (71.9%)^a^	75 (28.1%)
Exposure to toxic chemicals	229 (85.8%)^a^	38 (14.2%)
Menstruating women are at risk of anemia	181 (67.8%)^a^	86 (32.2%)
Intake of certain drugs	218 (81.6%)^a^	49 (18.4%)
Reduced intake of iron-containing diets	236 (82.4%)^a^	31 (11.6%)
Reduced intake of vitamin B12-containing diets	225 (84.3%)^a^	42 (15.7%)
Reduced intake of folate-containing diets	221 (82.8%)^a^	46 (17.2%)
*Knowledge of prevention of anemia*		
Frequent blood donation	107 (40.1%)	160 (59.9%)^a^
Ingesting less iron-rich foods	101 (37.8%)	166 (62.2%)^a^
Regular water drinking	214 (80.1%)^a^	53 (19.9%)
Regular intake of vitamin B12-rich diets	225 (84.3%)^a^	42 (15.7%)
Reduced intake of folate-rich diets	130 (48.7%)	137 (51.3%)^a^
Reduced intake of vitamin C-rich diets	107 (40.1%)	160 (59.9%)^a^
*Knowledge of the relationship between CKD and anemia*		
CKD has nothing to do with anemia	114 (42.7%)	153 (57.3%)^a^
Iron deficiency anemia is a common complication in CKD	197 (73.8%)^a^	70 (26.2%)
CKD patients with anemia usually have cognitive impairment	172 (64.4%)^a^	95 (35.6%)
CKD patients with anemia can also have high lipid levels in the blood	185 (69.3%)^a^	82 (30.7%)
CKD patients with anemia can also develop stroke	180 (67.4%)^a^	87 (32.6%)
CKD patients with anemia can also develop heart muscle disease	191 (71.5%)^a^	76 (28.5%)
CKD patients with anemia can also develop lung diseases	191 (71.5%)^a^	76 (28.5%)
Patients on dialysis can develop anemia	181 (67.8%)^a^	86 (32.2%)
Regular intake of iron-rich foods can help manage iron deficiency anemia in CKD patients	204 (76.4%)^a^	63 (23.6%)
Regular intake of vitamin-rich foods and folate-rich foods can help reduce the severity of anemia in CKD patients	213 (79.8%)^a^	54 (20.2%)

*Note:* The data is presented as frequencies and percentages, *n* (%).

Abbreviation: CKD, chronic kidney disease.

^a^Correct answer.

**Table 4:** Summary (scores) of general knowledge assessment of participants on CKD, anemia, and CKD–anemia interrelationship.

**Knowledge classifications**	**Mean score (SD)**	**Knowledge levels, number (%)**	
General knowledge of CKD, causes, risk factors, and preventive practices	23.19 (6.98)	Poor knowledge (scores < 23)	Good knowledge (scores 23–34)
		59 (22.1)	208 (77.9)
General knowledge of anemia, causes, risk factors, and preventive practices	23.42 (6.23)	Poor knowledge (scores < 23)	Good knowledge (scores 23–35)
		52 (19.5)	215 (80.5)
Relationship between CKD and anemia	5.27 (2.57)	Poor knowledge (scores < 5)	Good knowledge (scores 5–10)
		45 (16.9)	222 (83.1)

Abbreviations: CKD, chronic kidney disease; SD, standard deviation.

**Table 5:** Association between demographic factors and the knowledge level of participants on CKD.

**Demographics**	**Students' knowledge, ** **n** ** (%)**		**COR**	**p** ** value**	**AOR**	**p** ** value**
	**Poor**	**Good**	**(95% CI)**		**(95% CI)**	
Age groups				0.35		0.08
17–19	8 (3.0)	13 (4.8)	1		1	
20–23	38 (14.2)	112 (42.0)	0.55 (0.21, 1.43)	0.22	1.85 (0.50, 6.8)	0.35
24–27	15 (5.6)	61 (22.9)	0.40 (0.14, 1.14)	0.08	4.26 (0.99, 18.22)	0.07
≥ 28	4 (1.5)	16 (6.0)	1.63 (0.40, 6.63)	0.50	4.57 (0.77, 27.13)	0.09
Gender				0.14		0.107
Male	33 (12.4)	141 (52.8)	1		1	
Female	25 (9.4)	68 (25.4)	1.57 (0.87, 2.85)	0.14	0.36 (0.16, 0.84)	0.107
Level				0.07		< 0.001
First year	8 (3.0)	22 (8.2)	1		1	
Second year	7 (2.6)	16 (6.0)	1.20 (0.36, 4.00)	0.76	0.003 (0.00, 0.52)	< 0.001
Third year	10 (3.8)	72 (27.0)	0.38 (0.13, 1.09)	0.07	0.17 (0.03, 1.16)	0.050
Fourth year	34 (12.7)	98 (36.7)	0.95 (0.39, 2.34)	0.92	0.13 (0.002, 0.09)	< 0.001
Faculty				< 0.001		< 0.001
Health-related	18 (6.7)	127 (47.6)	1		1	
Nonhealth-related	41 (15.4)	81 (30.3)	3.57 (1.92, 6.64)	< 0.001	0.02 (0.00, 0.09)	< 0.001

*Note:p* value of ≤ 0.05 is statistically significant; *χ*^2^ (df) represents chi-square (degree of freedom).

Abbreviations: AOR, adjusted odds ratio; CI, confidence interval; COR, crude odds ratio.

**Table 6:** Association between demographic factors and the knowledge of participants on anemia, causes, risk factors, and preventive practices.

**Demographics**	**Students' knowledge, ** **n** ** (%)**		**COR**	**p** ** value**	**AOR**	**p** ** value**
	**Poor**	**Good**	**(95% CI)**		**(95% CI)**	
Age groups				0.001		0.012
17–19	11 (4.1)	10 (3.7)	1		1	
20–23	30 (11.2)	120 (45.0)	0.23 (0.09, 0.59)	0.001	15.21 (1.99, 116.38)	0.009
24–27	10 (3.8)	66 (24.7)	0.14 (0.05, 0.41)	< 0.001	30.82 (3.35, 283.59)	0.002
≥ 28	5 (1.9)	15 (5.6)	0.30 (0.08, 1.14)	0.07	47.26 (4.21, 530.21)	0.002
Gender				0.62		0.062
Male	33 (12.4)	141 (52.8)	1		1	
Female	20 (7.4)	73 (27.3)	1.17 (0.63, 2.18)	0.62	0.26 (0.07, 0.95)	0.062
Level				0.07		0.110
First year	11 (4.1)	19 (7.1)	1		1	
Second year	7 (2.6)	16 (6.0)	0.76 (0.24, 2.41)	0.64	0.19 (0.04, 1.01)	0.052
Third year	12 (4.5)	70 (26.2)	0.30 (0.11, 0.78)	0.01	0.77 (0.21, 2.81)	0.690
Fourth year	31 (11.6)	101 (37.9)	0.53 (0.23, 1.23)	0.14	0.40 (0.12, 1.37)	0.150
Faculty				< 0.001		< 0.001
Health-related	16 (6.0)	129 (48.3)	1		1	
Nonhealth-related	36 (13.5)	86 (32.2)	3.38 (1.76, 6.46)	< 0.001	0.01 (0.00, 0.007)	< 0.001

*Note:p* value ≤ 0.05 is statistically significant, whereas *χ*^2^ (df) is chi-square (degree of freedom).

Abbreviations: AOR, adjusted odds ratio; CI, confidence interval; COR, crude odds ratio.

## Data Availability

All data will be made available to interested parties upon sending a request to the corresponding author.
